# Magnetite graphene oxide-albumin conjugate: carrier for the imatinib anticancer drug

**DOI:** 10.1007/s10856-023-06735-1

**Published:** 2023-07-14

**Authors:** Maral Mashreghi, Bahare Sabeti, Fereshteh Chekin

**Affiliations:** 1 Department of Pharmacy, Ayatollah Amoli Branch, Islamic Azad University, Amol, Iran; 2 Department of Chemistry, Ayatollah Amoli Branch, Islamic Azad University, Amol, Iran

## Abstract

Carbon nanomaterials are widely used in biomedical applications due to their versatile properties. These are the attractive candidates for the carrying of anticancer drugs, genes, and proteins for chemotherapy. Imatinib is an effective chemotherapy drug whose toxicity has created a significant limitation in treatment. In this research, a new biocompatible nanocarrier based on albumin-magnetite graphene oxide conjugates was reported for the loading and release of imatinib. The magnetite graphene oxide nanocomposite was investigated by ultra violet-visible spectroscopy (UV-Vis), field emission scanning electron microscope (FE-SEM), X-ray diffraction spectroscopy (XRD) and energy diepersive X-ray spectroscopy (EDX) methods. The crystallite size of Fe_3_O_4_ nanoparticles on graphene oxide obtained from XRD is about 14 nm which is in agreement well with the SEM results. We show that magnetite graphene oxide conjugated with albumin is an extremely efficient carrier. An efficient loading of IM, 81% at pH 7.0, time 2 h and initial concentration of 1 mg/mL was seen onto magnetite graphene oxide-albumin in comparison to graphene oxide and magnetite graphene oxide due to the presence of oxygen and nitrogen functional groups of albumin. Upon the pH 9.0 and 7.0, 7% and 16% imatinib could be released from the magnetite graphene oxide-albumin in a time span of 5 h but when exposed pH 4.0 the corresponding 31% was released in 5 h. After 20 h, 21, 42 and 68% of imatinib was released at pH 9.0, 7.0 and 4.0, respectively. This illustrates the major benefits of the developed approach for biomedical applications.

**Graphical Abstract:**

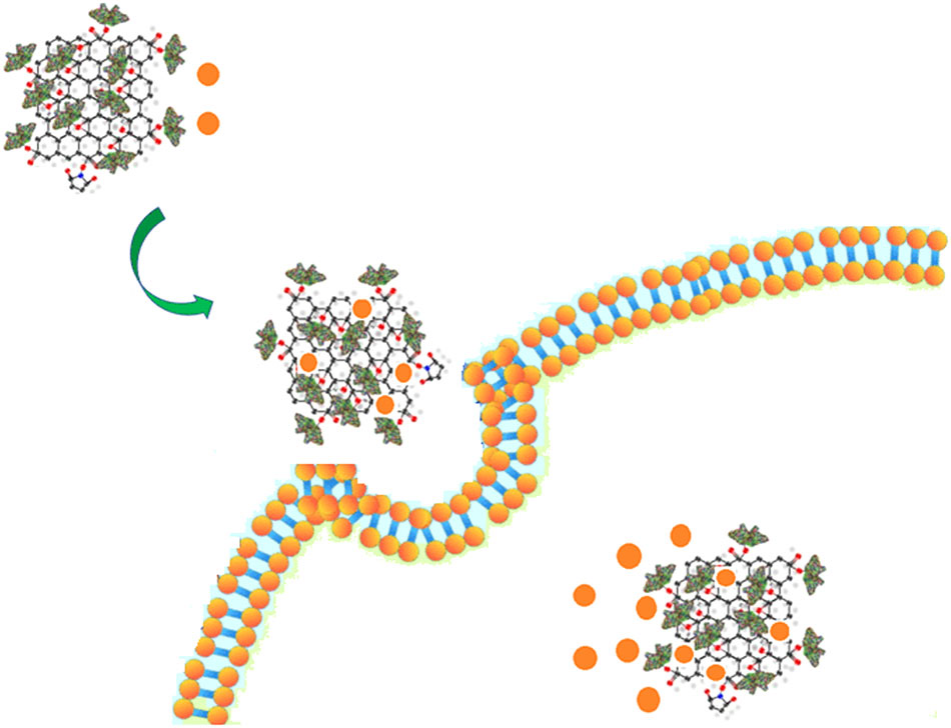

## Introduction

Rapid progress in drug discovery methods has caused an exponential increase in new drugs. Due to the various physical and chemical properties of different drugs, we need more intelligent drug delivery systems [1]. The purpose of any drug delivery system is to provide the required amount of drug to the right place in the body by quickly reaching it and maintaining the maintenance dose of the drug [2, 3]. With the progress of medical science, it seems that traditional drug delivery systems need to be modified and changed to improve the quality of drug delivery and reduce the toxicity of drugs [4, 5]. One of the most critical categories in the discussion of drug delivery is the controlled release in the body, which in the traditional drug delivery system practically has no control over the time, place, and speed of the drug release. In addition, the drug concentration is regularly in the circulating blood [6]. By using new drug delivery systems, also called controlled release drug delivery systems, three areas of drug release speed, time, and place can be controlled and determined [7]. Targeted drug delivery is an excellent way to develop the effectiveness and health of therapeutic agents. In the field of cancer treatment, drug delivery systems play a significant role in increasing the effectiveness of treatment. With the advancement of technology, new methods were invented to solve this problem [8, 9].

With the current rapid progress in nanotechnology, progress in nanomaterials engineering has created many hopes in the design and use of drug carriers, so these new drug carriers create much potential in improving drug packaging, transport, and efficiency in targeting [10]. The drug carrier should be selected and designed in such a way that after the release of the drug from the carrier, it will be destroyed in the body after a while; otherwise, it will not cause any harm to the human body [11]. At the same level as other nanocarriers, graphene has four times more ability to transport and carry drugs. Another essential feature of graphene and its derivatives in drug delivery is the loading ratio, which can increase up to 200% in the case of graphene nanomaterials, which is significantly higher than other nanomaterials and other drug delivery systems [12, 13]. The structure’s unique two-dimensional shape and flatness, large chemical surface, high chemical and mechanical stability, low cytotoxicity, and good biocompatibility of graphene and graphene oxide, and the absence of this shape in the morphology of the biological system of the human body are another advantage for its use [14]. Graphene oxide is hydrophilic and can be dispersed in water as a stable colloid [15]. Graphene oxide and its derivatives have increased biocompatibility by having hydrophilic epoxy, hydroxyl, and acidic groups [16].

Proteins are complex biopolymers with hydrophobic and hydrophilic segments, that may serve as an adhesive for solid surfaces [17]. Bovine serum albumin (BSA) is a spherical shape protein which has 583 amino acid residues [18]. In the structure of BSA, there is tyrosine residues which distinguishes it as a distinct reducing agent. Hydrophobic sections of BSA may be adsorbed to hydrophobic surface area, whereas hydrophilic parts of BSA could be interacted with water functional groups in the presence of oxygen [19].

Imatinib, under the brand name Glivec, is used to treat certain types of leukemia, acute lymphoblastic leukemia, bone marrow disorders, skin cancer, or certain tumors of the stomach and digestive tract [20, 21]. Common side effects of imatinib include vomiting, diarrhea, muscle pain, headache, and skin rash. More severe complications include gastrointestinal bleeding, bone marrow failure, liver problems, and heart failure. This drug works by inhibiting tyrosine-kinase, leading to reduced cell growth or apoptosis in some cancer cells. It is now part of the essential drugs of the World Health Organization, which are a collection of the most effective drugs in the health system [22].

In this research, an attempt is made to synthesize magnetite graphene oxide-albumin conjugate by a simple and easy method. Then by preparing a nanostructure of magnetite graphene oxide with albumin, it tries to load an imatinib anticancer drug on this nanostructure as a nanocarrier. Moreover, the effect of different factors such as time and pH was investigated on the loading and releasing.

## Experimental

### Chemicals

Graphene oxide was purchased from Iranian Nano Materials Pioneers. Bovine serum albumin, iron(II) sulfate hepta hydrate, phosphoric acid, sodium dihydrogen phosphate, disodium hydrogen phosphate, sodium phosphate, and imatinib were purchased from Sigma-Aldrich and used as received.

### Apparatus

FE-SEM images were obtained using an electron microscope (MIRATESCAN-XMU, Czech Republic) combined with EDS (energy-dispersive X-ray Spectroscopy) machine. X-ray diffraction measurement was recorded on a Bruker D8-Advance X-ray diffractometer (Germany). Electrochemical measurements were performed with a potentiostat/galvanostat (Sama 500-c Electrochemical Analysis system, Sama, Iran). A conventional three-electrode configuration consisting of Ag|AgCl|KCl3M as the reference electrode, a platinum wire as auxiliary electrode and CPE and IM@Fe3O4-GO-BSA modified CPE as working electrodes was employed. UV-Vis analysis of samples was recorded by UV-Vis spectrophotometer (UV-1900, Shimadzu Co., Japan).

### Preparation of magnetite graphene oxide-albumin nanostructure

The magnetite graphene oxide was prepared based on a work by Vatandost [23]. For the synthesis of magnetite graphene oxide, 10 mg of graphene oxide powder was dispersed in 10 mL of distilled water for 30 min by ultrasonic bath. 10 mL of, iron(II) sulfate hepta hydrate solution (0.5 M) was added to the dispersed graphene oxide solution with vigorous stirring, and the pH of the solution was adjusted to 10 by NaOH solution. The solution was transferred to a steel container and heated in an autoclave for 8 h at a temperature of 180 °C. Finally, the product (Fe_3_O_4_-GO) was washed with water and ethanol and dried overnight in an oven at 60 °C.

For magnetite graphene oxide-albumin (Fe_3_O_4_-GO-BSA) composite synthesis, 3 mg of magnetite graphene oxide powder was dispersed in 30 mL of distilled water for 30 min in an ultrasonic bath. Then 10 mL of BSA solution (0.5 mg/mL) was added to the dispersed solution along with stirring was added and the mixture was stirred at room temperature for 2 h. Finally, the suspension solution was filtered and washed with water. In the end, the product was dried and stored in the refrigerator for use.

### Imatinib calibration curve

The behavior of imatinib was studied in distilled water by UV-Vis spectroscopy, and the λ_max_ of imatinib was determined to be 242 nm. Then, the absorption of imatinib solutions with different concentrations was recorded at 242 nm wavelength to draw the imatinib calibration graph.

### Loading and on-demanded release experiments

1 mg of Fe_3_O_4_-GO-BSA nanocomposite in 4 mL phosphate buffer solution (PBS, 0.1 M) with different pH was dispersed for 30 min at 25 °C. 0.5 mg of IM was added to Fe_3_O_4_-GO-BSA dispersed solution and continuously stirred for 2 h. The product was centrifuged with relative centrifugal force of 8000 g for 15 min. The collected IM@Fe_3_O_4_-GO-BSA washed with water and dried overnight at room temperature. The loaded percent of IM on Fe_3_O_4_-GO-BSA nanocomposite was determined based on the standard curve of IM (absorbance vs. concentration) at 242 nm with the concentration difference of IM between the initial IM solution (C_0_) and the supernatant solution (C_s_) using the following equation [24, 25]:

$${{{\mathrm{Loading}}}}\,\% = \left[ {\left( {{{{\mathrm{C}}}}_0-{{{\mathrm{C}}}}_{{{\mathrm{s}}}}} \right)/{{{\mathrm{C}}}}_0} \right] \times 100$$

The on-demand release was done by dialysis tubing. 1 mg of IM@Fe_3_O_4_-GO-BSA was dispersed in 1 mL of PBS (0.1 M), placed into dialysis tubing and dialyzed in 10 mL of PBS solution with pH 4.0 and 7.0. The released% of IM was calculated with recording absorbance of PBS at 245.

### Preparation of modified electrode

The carbon paste electrode (CPE) was prepared based on previous report [26, 27]. The graphite powder plus paraffin hand-mixed until a uniformly wetted paste was obtained. Then the carbon paste was packed into a glass tube (with internal radius 3 mm). Electrical contact was made by a copper wire. The new surface of electrode was obtained by polishing it on a weighing paper. 1 mg of IM@Fe_3_O_4_-GO-BSA was added to 1 mL of water and sonicated for 30 min. 5 µL of this solution was drop-casted onto the CPE and allowed to dry at room temperature.

## Results and discussions

### Characterization

**Fig. 1:**
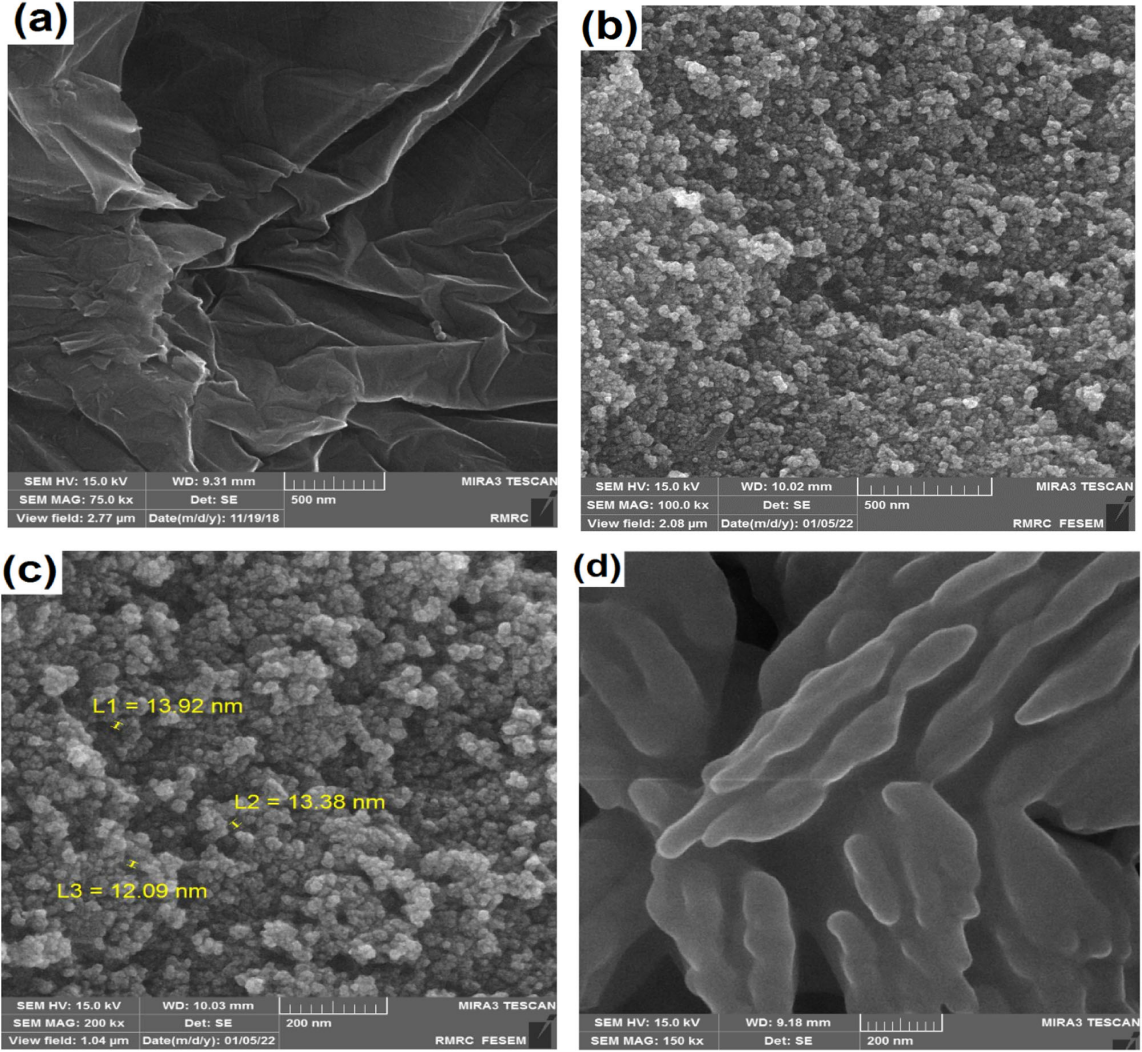
FE-SEM images of (**a**) GO, (**b**) and (**c**) Fe_3_O_4_-GO with different magnifications, (**d**) IM@ Fe_3_O_4_-GO-BSA

The morphology of GO, Fe_3_O_4_-GO, and IM@Fe_3_O_4_-GO-BSA is characterized with FE-SEM. As can be seen, GO has a relatively smooth and wavy surface and has thin layers (Fig. 1a). In contrast, the image of Fe_3_O_4_-GO (Fig. 1b) shows that GO sheets is decorated by sphere shape Fe_3_O_4_ nanoparticles with diameter 12–14 nm (Fig. 1c). This observation confirms the formation of Fe_3_O_4_-GO. While stacking and protuberances are observed on the surface of IM@Fe_3_O_4_-GO-BSA (Fig. 1d), obviously indicating IM immobilized onto the Fe_3_O_4_-GO-BSA composite. The different functional groups such as phenolic, ketone, lactone, carboxyl, quinone, epoxy and amine on Fe_3_O_4_-GO-BSA nanocomposite form hydrogen interaction with functional groups of IM. Moreover, there are the π-π stacking interaction as well as the hydrophobic effect between IM and Fe_3_O_4_-GO-BSA composite.

**Fig. 2:**
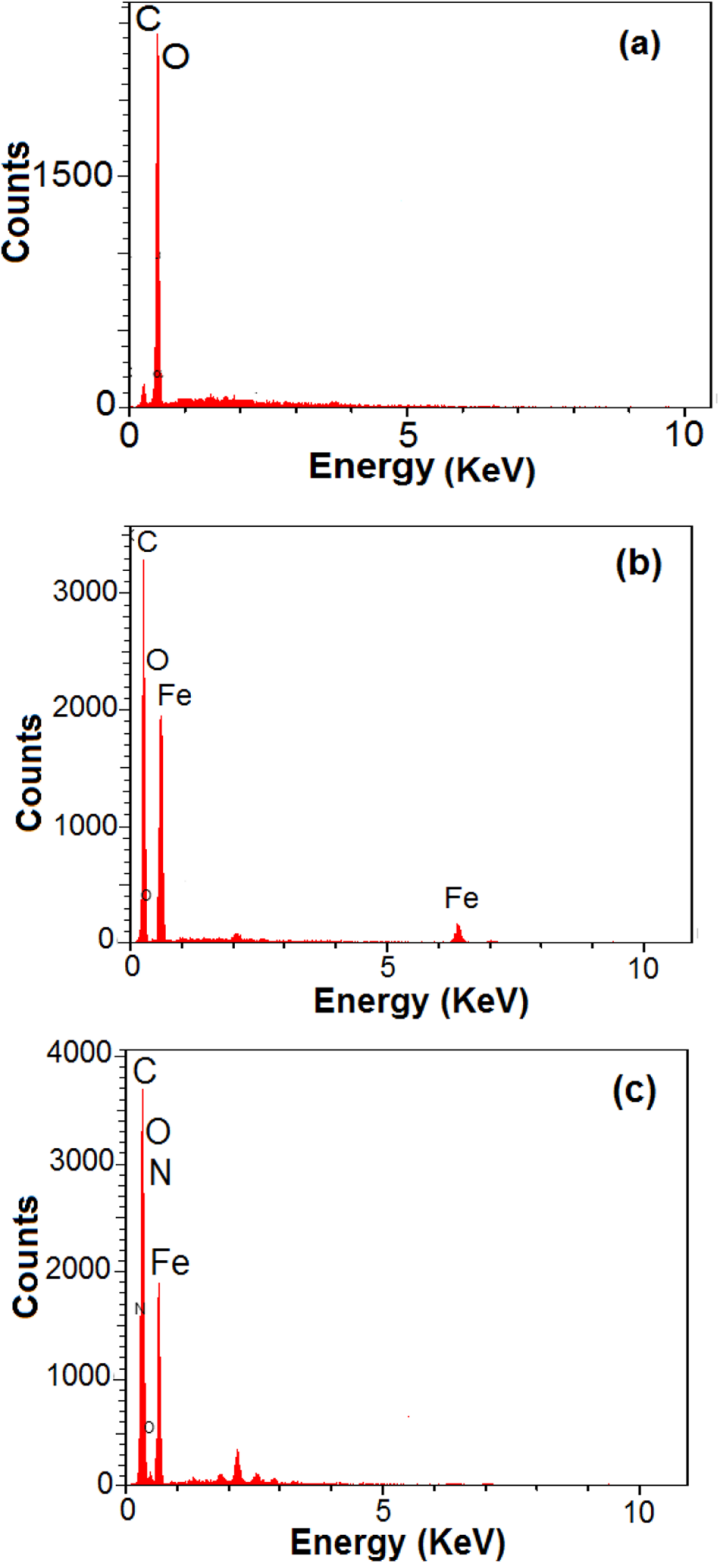
EDX spectra of (**a**) GO, (**b**) Fe_3_O_4_-GO, and (**c**) IM@Fe_3_O_4_-GO-BSA

EDX is an analysis method used for the structural analysis of the chemical properties of a sample. This method is generally based on the principle that each element has a unique atomic structure that enables a set of peaks in its X-ray spectrum. Figure 2 shows the EDX spectrum of GO, Fe_3_O_4_-GO, and IM@Fe_3_O_4_-GO-BSA. In the spectrum of GO, only C and O elements are seen, in the spectrum of Fe_3_O_4_-GO, in addition to C and O elements, the presence of Fe element (60.81%) is proof of the synthesis of Fe_3_O_4_-GO, while in the spectrum of IM@Fe_3_O_4_-GO-BSA, the presence of N element confirms the loading of IM on Fe_3_O_4_-GO-BSA composite.

**Fig. 3:**
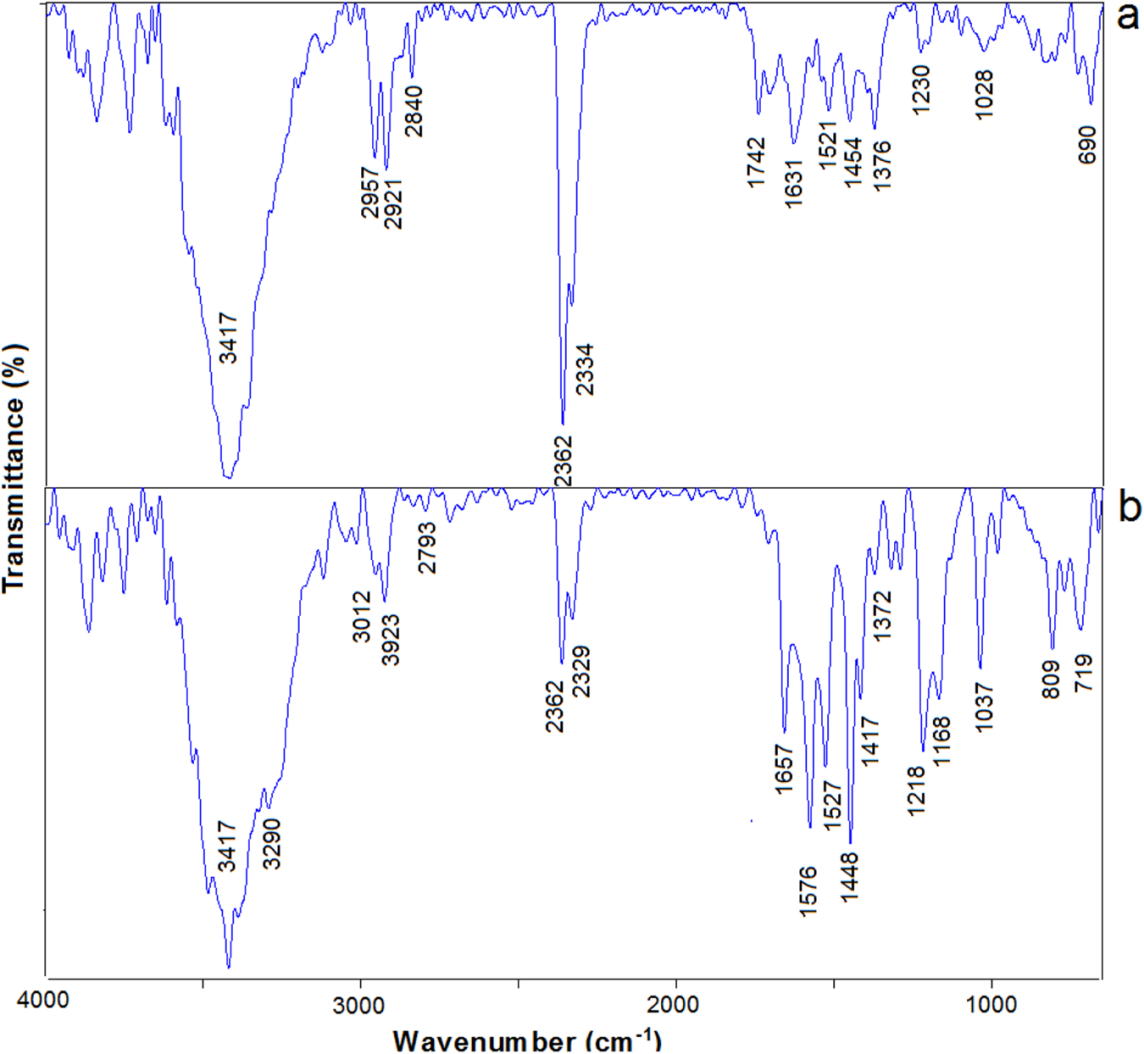
FT-IR spectra of (**a**) Fe_3_O_4_-GO and (**b**) IM@Fe_3_O_4_-GO-BSA

Figure 3 shows the comparison FT-IR data of Fe_3_O_4_-GO and IM@Fe_3_O_4_-GO-BSA. In the spectrum Fe_3_O_4_-GO, the presence of peaks at 1029 cm^−1^ (C-O-C stretching vibration of epoxide group), 1230 cm^−1^ (C-OH), 1376 cm^−1^ (C-O asymmetric stretching vibration of carboxylic group), 1631 cm^−1^ (C=C in the carbon skeletal network), 1742 cm^−1^ (C=O stretching vibration of carboxylic group), 2362 cm^−1^ (CO_2_), 2840 cm^−1^ (CH bending vibration), 2921 cm^−1^ (CH stretching vibration) is attributed to GO and the strong peak around 3417 cm^−1^ can ascribe to the O-H stretching mode of water molecules. The existence of peaks at 690, 1454, 1521, 2334, 2957 cm^−1^ is ascribed to the presence of Fe_3_O_4_ nanoparticles. The infrared spectrum of the IM@Fe_3_O_4_-GO-BSA showed characteristic bands for IM and Fe_3_O_4_-GO-BSA compounds. The intense infrared absorption bands for IM appeared at 2932 cm^−1^ (C-H stretching N-methylpiperazine ring vibrations), 1657 cm^−1^ (C=O stretching vibration mixed with C=O rocking vibration and C-N stretching vibration), 1576 cm^−1^ (C-C and C-N stretching pyridine and aminopyrimidine ring vibrations mixed with in-plane deformation of C-H), 1448 cm^−1^ (C-H symmetric and asymmetric deformations of the N-methylpiperazine ring), 1417 cm^−1^ (in-plane deformation of C-H mixed with stretching vibration of C-N of pyridine and aminopyrimidine rings, as well as C-C stretching methylbenzene ring vibration), and 809 cm^−1^ (out-of-plane bending mixed with asymmetric torsion of the methylbenzene ring). The band observed at 3290 cm^−1^ was associated with the N–H stretching vibration of open-chain amides in the imatinib solid state.

**Fig. 4:**
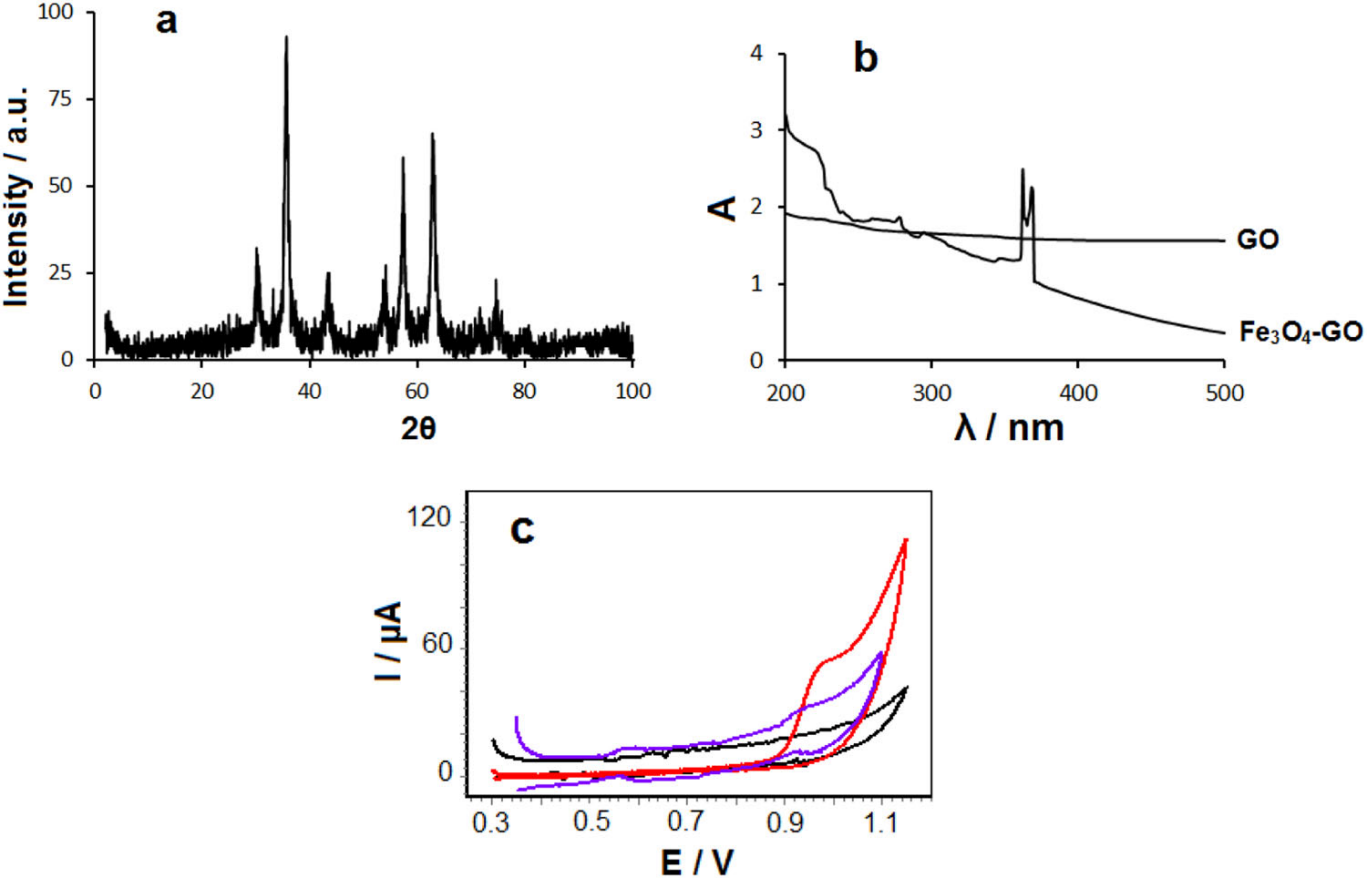
**a** XRD spectrum of Fe_3_O_4_-GO; **b** UV-Vis spectra of GO and Fe_3_O_4_-GO; **c** cyclic voltammograms of unmodified CPE (black curve), 0.1 mM IM at CPE (red curve) and IM@ Fe_3_O_4_-GO-BSA modified CPE (violet curve) in 0.1 M PBS (pH 7.0) at 50 mV s^−1^

Powder X-ray diffraction (XRD) is an effective method to investigate the inter layer changes and the crystalline properties of synthesized samples. Figure 4a shows the XRD pattern of Fe_3_O_4_-GO sample. As seen, the diffraction peaks at 30.12, 35.64, 43.64, 54.16, 57.36, 63.04, and 74.64, which corresponded to the (220), (311), (400), (422), (511), (440), and (533) lattice planes of the face-centered cubic (fcc) spinel phase of Fe_3_O_4_ (JCPDS:19-0629), respectively [28]. However, the peak of GO can not be seen, which may be due to the low contents of GO [29]. The particles size was calculated from the XRD data using Scherrer’sequation [30]:

$${{{\mathrm{D}}}} = {{{\mathrm{k}}}}\,\lambda /\beta \,{{{\mathrm{cos}}}}\,\theta$$

where D is particle size, k is the grain shape factor taken as unity contemplating that the particles are spherical in shape, λ is the incident x-ray wavelength of Cu–Kα radiation and θ is the Bragg’s angle, β is the broadening of diffraction line measured at half maximum intensity (radians). The determined particle size came out to be 14.38 nm which is in agreement well with the SEM results.

The evidence of formed Fe_3_O_4_-GO hybrid is characterized by UV-Vis spectroscopy in the range of 200–500 nm (Fig. 4b). The GO shows any absorption peak at this range, while the Fe_3_O_4_-GO shows the absorption peaks at 362 and 369 nm in good agreement with Fe_3_O_4_ nanoparticles; thus the UV-vis spectra confirm the formed Fe_3_O_4_-GO hybrid.

### Electrochemical behavior of IM@Fe_3_O_4_-GO-BSA

The cyclic voltammetry was used for investigating the formation of IM@Fe_3_O_4_-GO-BSA hybrid (Fig. 4c). IM at surface of CPE shows oxidation peak at 0.92 V (red curve) in 0.1 M PBS (pH 7.0), while IM@Fe_3_O_4_-GO-BSA/CPE (violet curve) shows oxidation peak at 0.90 V. It is found that the interactions (hydrogen binding and π-π stacking) between Fe_3_O_4_-GO-BSA and IM facilities electron transfer.

### UV-Vis spectroscopy

**Fig. 5:**
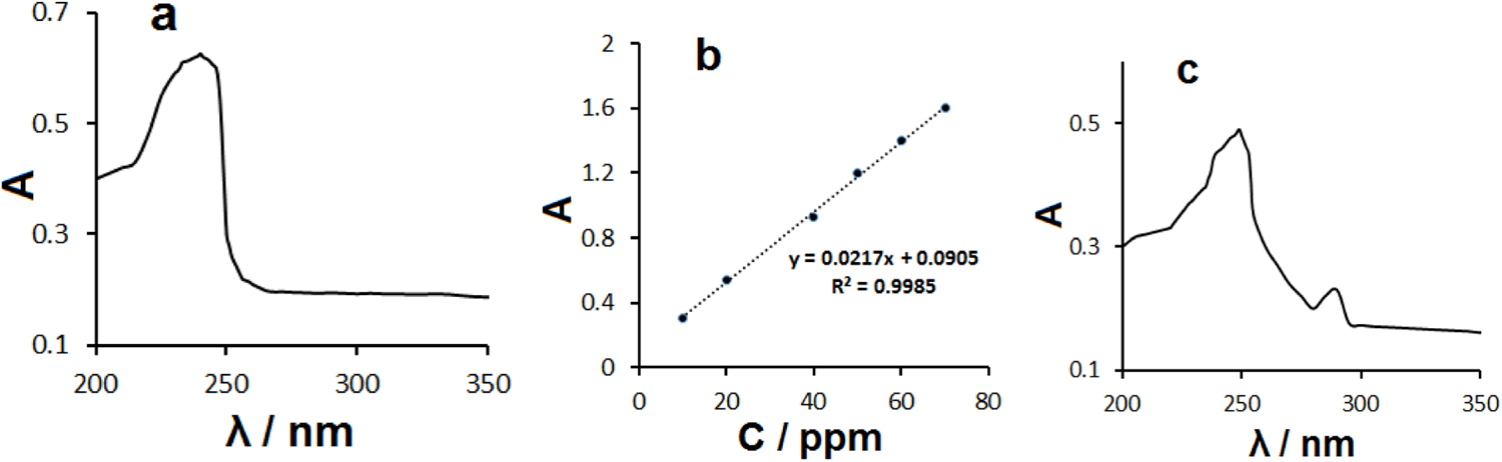
**a** UV-Vis spectrum of 25 ppm IM; **b** The calibration curve of IM absorbance to its concentration; **c** UV-Vis spectrum of IM@Fe_3_O_4_-GO-BSA (2 mg/mL)

The loading capacity of Fe_3_O_4_-GO-BSA for IM was determined from UV-Vis analysis (Fig. 5a) based on standard curve of IM absorbance to its concentration between 10–70 ppm at 242 nm according to A = 0.0905 + 0.0217 × [IM] (ppm) with a correlation coefficient of 0.9985 (Fig. 5b).

The confirmation of the formation of IM@Fe_3_O_4_-GO-BSA is determined by UV-Vis spectroscopy in the range of 200–350 nm (Fig. 5c). The Fe_3_O_4_-GO-BSA hybrid shows characteristic the absorption peaks at 248 and 290 nm in good agreement with IM and BSA, respectively; thus the UV-vis spectrum confirm the formed IM@Fe_3_O_4_-GO-BSA hybrid. The absorption peak of the free IM is shown at 242 nm (Fig. 5a) that after hybridized with Fe_3_O_4_-GO-BSA shifted towards the red part due to the ground-state electron donor acceptor interaction between IM and Fe_3_O_4_-GO-BSA.

### Amount of loaded IM on different carriers

**Fig. 6:**
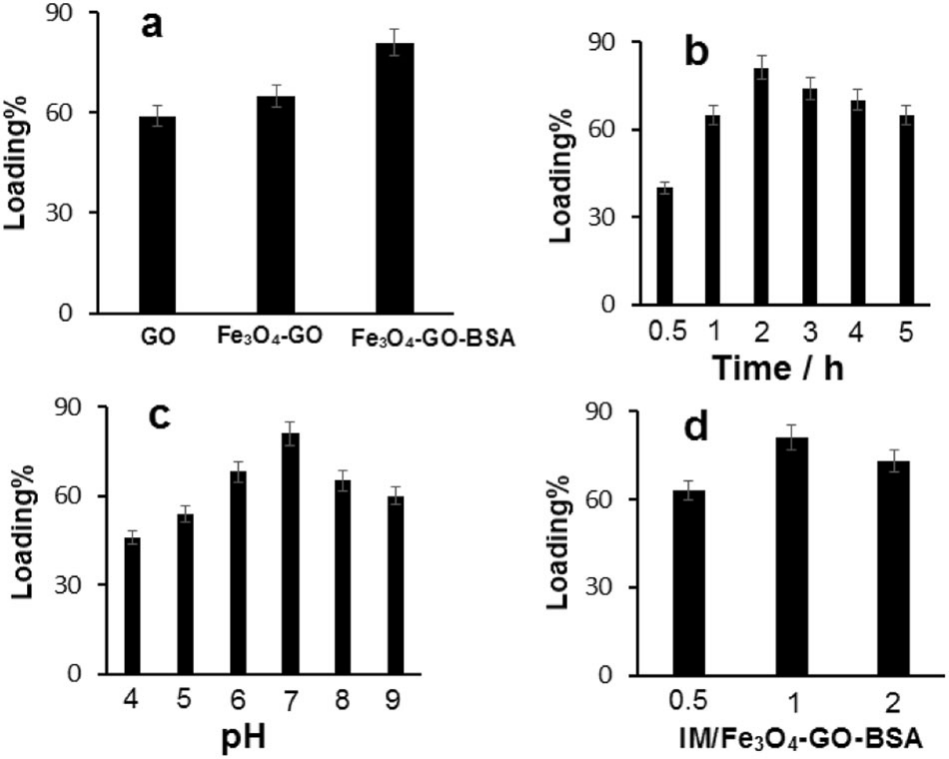
**a** Comparison of the loaded IM percent onto GO, Fe_3_O_4_-GO, and Fe_3_O_4_-GO-BSA; **b** Effect of shaking time on loading percent; **c** The pH effect of PBS solution on loading percent; **d** Effect of IM/Fe_3_O_4_-GO-BSA ratio on loading percent

The percentage of loaded IM on GO, Fe_3_O_4_-GO, and Fe_3_O_4_-GO-BSA carriers in PBS (0.1 M, pH = 7.0) at room temperature for 2 h was shown in Fig. 6a. As seen, the amount of loaded IM on the Fe_3_O_4_-GO-BSA nanocomposite is more than that of GO and Fe_3_O_4_-GO. This result implies that Fe_3_O_4_-GO-BSA makes stronger hydrogen binding with IM due to the presence of oxygen and nitrogen functional groups of BSA.

### Optimization of effective parameters in IM loading

The IM content loaded onto Fe_3_O_4_-GO-BSA was controlled by setting the different shaking time and pH of PBS solution during the loading tests. As can be seen in Fig. 6b, 2 h time was chosen as optimum time for loading of IM and further studies were performed at this time. This result shows that within 2 h, the surface of Fe_3_O_4_-GO-BSA is saturated with IM. The pH effect of the PBS solution on loaded percent of IM at room temperature and shaking time of 2 h was investigated and the results are shown in Fig. 6c. The results showed that the natural medium is favorable for loading IM. This may be due to strongest hydrogen bonding interaction of –OH, –NH_2_ and –COOH of Fe_3_O_4_-GO-BSA with nitrogen and oxygen functional groups of IM. In the research carried out by Hajir et al. on loading the doxorubicin anticancer drug on the porous reduced graphene oxide/chitosan hybrid, pH 7.0 was reported as the optimal pH for loading (24). The loading of IM drug in PBS (0.1 M, pH 0.7) was recorded at 2 h with different ratios of IM to Fe_3_O_4_-GO-BSA. As shown in Fig. 6d, the amount of IM loading has increased from 0.5 to 1. Therefore, the 1:1 is the best ratio for IM loading on Fe_3_O_4_-GO-BSA.

### IM release from IM@Fe_3_O_4_-GO-BSA

**Fig. 7:**
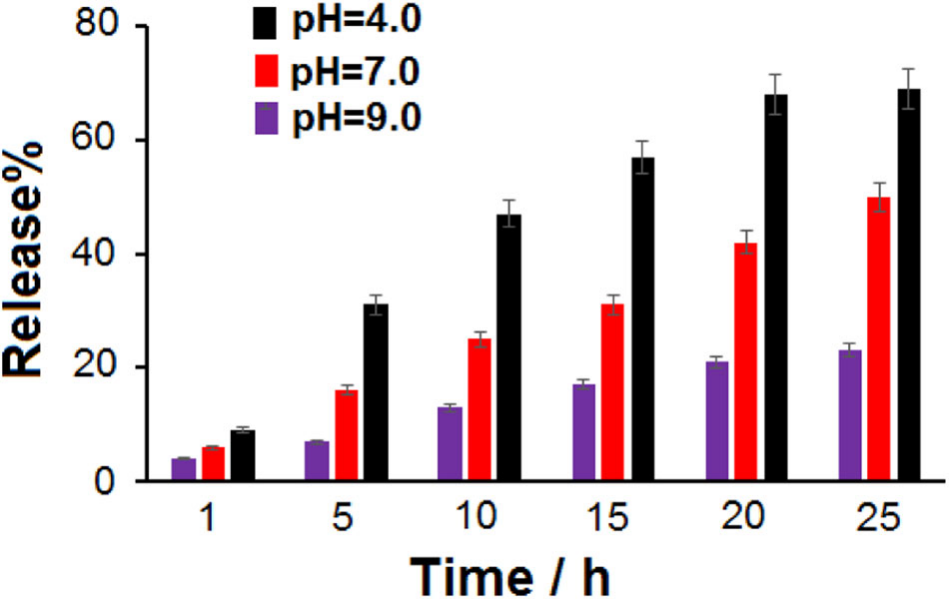
The release percent of IM from IM@Fe_3_O_4_-GO-BSA in PBS solution with pH 4.0, 7.0, and 9.0 at different times

Figure 7 shows the release of IM from IM@Fe_3_O_4_-GO-BSA in 0.1 M PBS at different pH and times. As shown, after 5 h at pH 9.0 and 7.0, 7 and 16% of IM are released from IM@Fe_3_O_4_-GO-BSA, while at pH 4.0, 31% of IM is released. As explained in the pH effect on IM loading, the strong hydrogen bond between IM and Fe_3_O_4_-GO-BSA at pH 7.0 is the reason for the low release of IM from the Fe_3_O_4_-GO-BSA surface. Also, in acidic pH, the release of IM is higher than in a neutral environment because IM is protonated in an acidic environment and its solubility increases. After 20 h, 21, 42, and 68% of IM are released at pH 9.0, 0.7, and 0.4, respectively.

## Conclusions

We have effectively designed a drug delivery nanocarrier based on magnetite GO and BSA conjugate for imatinib anticancer drug. The properties of Fe_3_O_4_-GO-BSA conjugate were characterized in detail by different spectroscopy and voltammetry methods. The crystallite size of Fe_3_O_4_ nanoparticles on graphene oxide obtained from XRD was about 14 nm which is in agreement well with the SEM results. Also, the results showed that albumin conjugated with magnetite graphene oxide provides an extremely efficiency in loading and release of IM. These in vitro results highlight the potential of BSA conjugated with magnetite GO for successful in vivo injection. The present findings showed that IM-loaded Fe_3_O_4_-GO-BSA can act as injectable drug depots that sustain IM release after intratumoral injection.
